# Supplementary figures and images for: Targeted metabolomics reveals the association between central carbon metabolism and pulmonary nodules
Source: PLoS One. 2023 Dec 7;18(12):e0295276. doi: 10.1371/journal.pone.0295276 (PMC10703222; doi:10.1371/journal.pone.0295276)

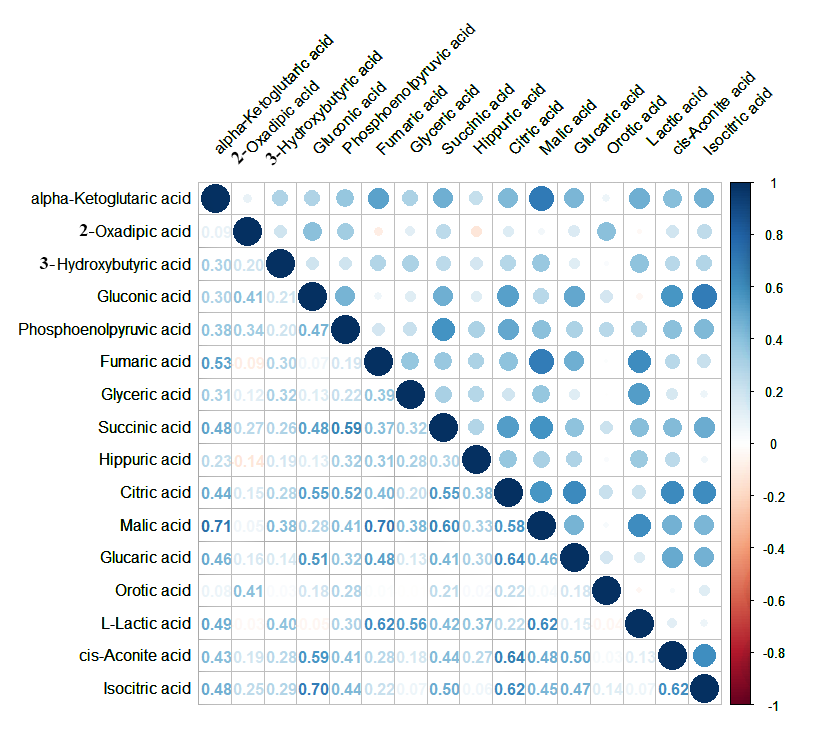


**S1 Fig. Spearman correlation coefficients between metabolites.**

Supplement: S1 Fig — (DOCX) [file pone.0295276.s001.docx]
