# Supplementary material for: Targeted metabolomics reveals the association between central carbon metabolism and pulmonary nodules
Source: PLoS One. 2023 Dec 7;18(12):e0295276. doi: 10.1371/journal.pone.0295276 (PMC10703222; doi:10.1371/journal.pone.0295276)
Supplement: S2 Fig — (PPTX) [file pone.0295276.s002.pptx]

## Slide 1
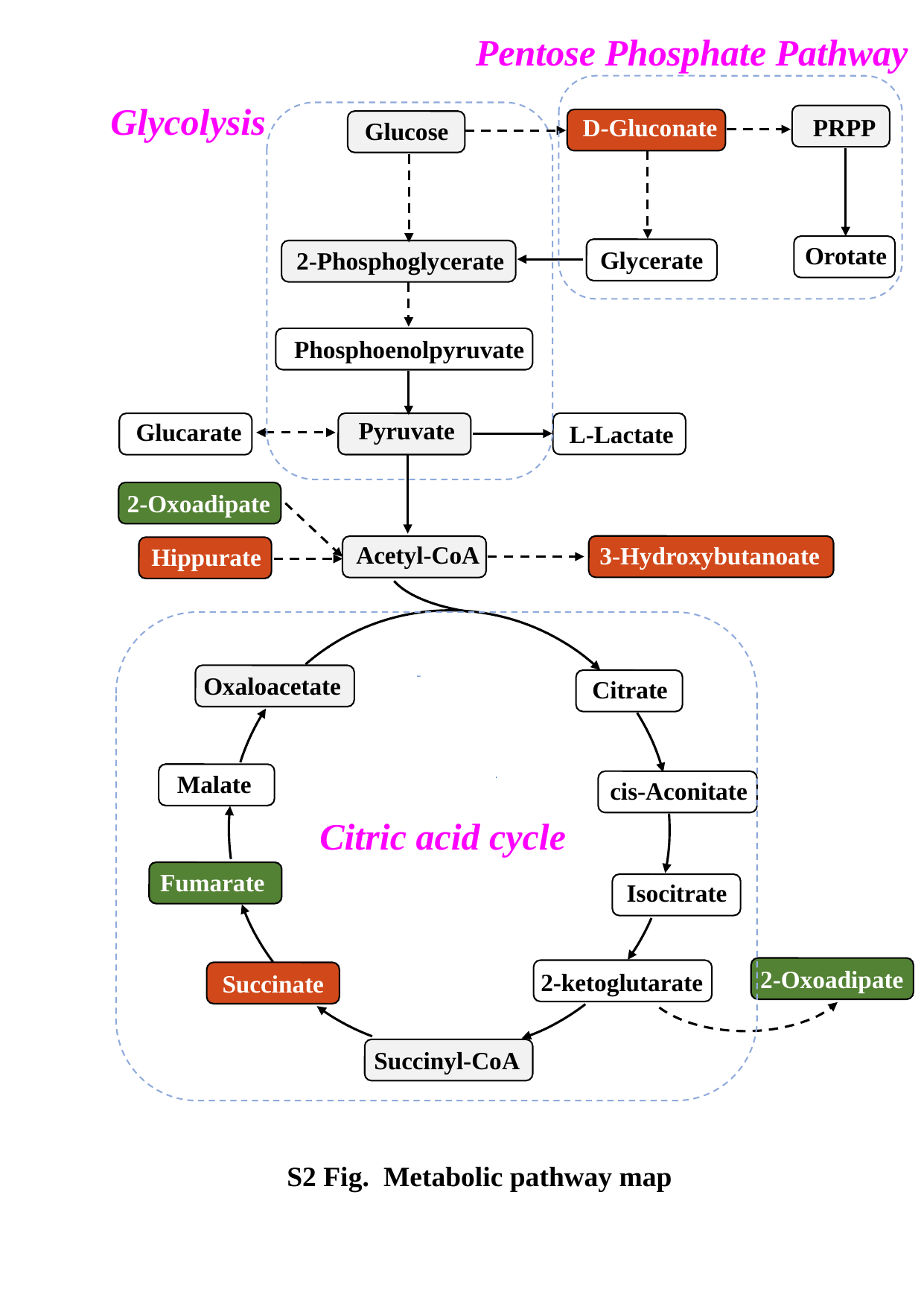

Pentose Phosphate Pathway
Glycolysis
PRPP
D-Gluconate
Glucose
Orotate
Glycerate
2-Phosphoglycerate
Phosphoenolpyruvate
Pyruvate
Glucarate
L-Lactate
2-Oxoadipate
Acetyl-CoA
3-Hydroxybutanoate
Hippurate
Oxaloacetate
Citrate
Malate
cis-Aconitate
Citric acid cycle
Fumarate
Isocitrate
2-Oxoadipate
2-ketoglutarate
Succinate
Succinyl-CoA
S2 Fig. Metabolic pathway map
