# Supplementary material for: Targeted metabolomics reveals the association between central carbon metabolism and pulmonary nodules
Source: PLoS One. 2023 Dec 7;18(12):e0295276. doi: 10.1371/journal.pone.0295276 (PMC10703222; doi:10.1371/journal.pone.0295276)
Supplement: S3 Fig — (DOCX) [file pone.0295276.s003.docx]

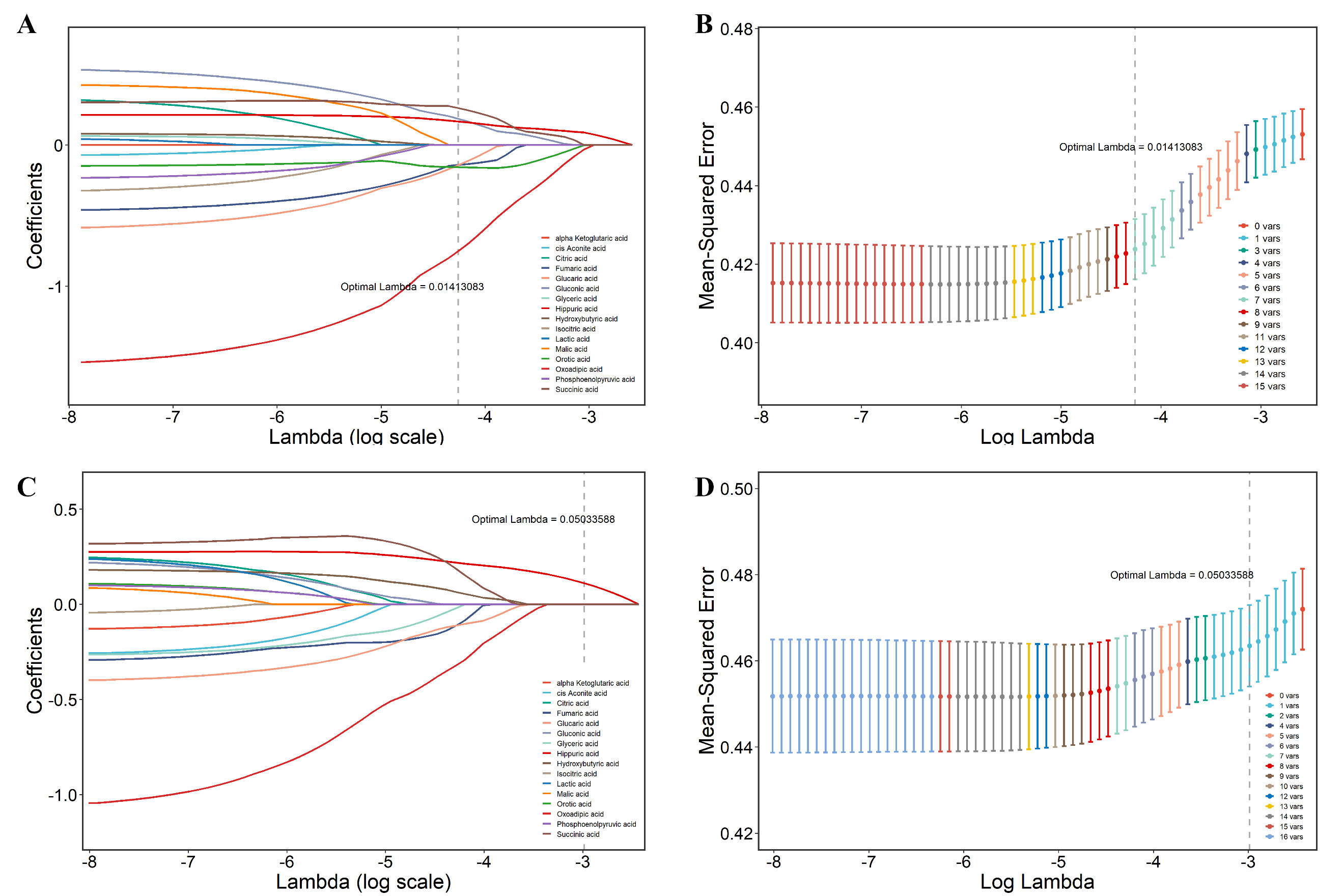


**S3 Fig.** **The metabolites selected into the multi-metabolite model by LASSO regression in subgroups stratified by age.** (A)(C) The LASSO solution path, with the coefficient profiles for 16 plasma metabolites as a function of the penalty parameter (log lambda). (B)(D) The prediction error of the LASSO regression model in function of the penalty parameter (log lambda).

(A)(B) Age<65years, (C)(D) Age ≥65years.
