# Supplementary material for: Targeted metabolomics reveals the association between central carbon metabolism and pulmonary nodules
Source: PLoS One. 2023 Dec 7;18(12):e0295276. doi: 10.1371/journal.pone.0295276 (PMC10703222; doi:10.1371/journal.pone.0295276)
Supplement: S5 Fig — (DOCX) [file pone.0295276.s005.docx]

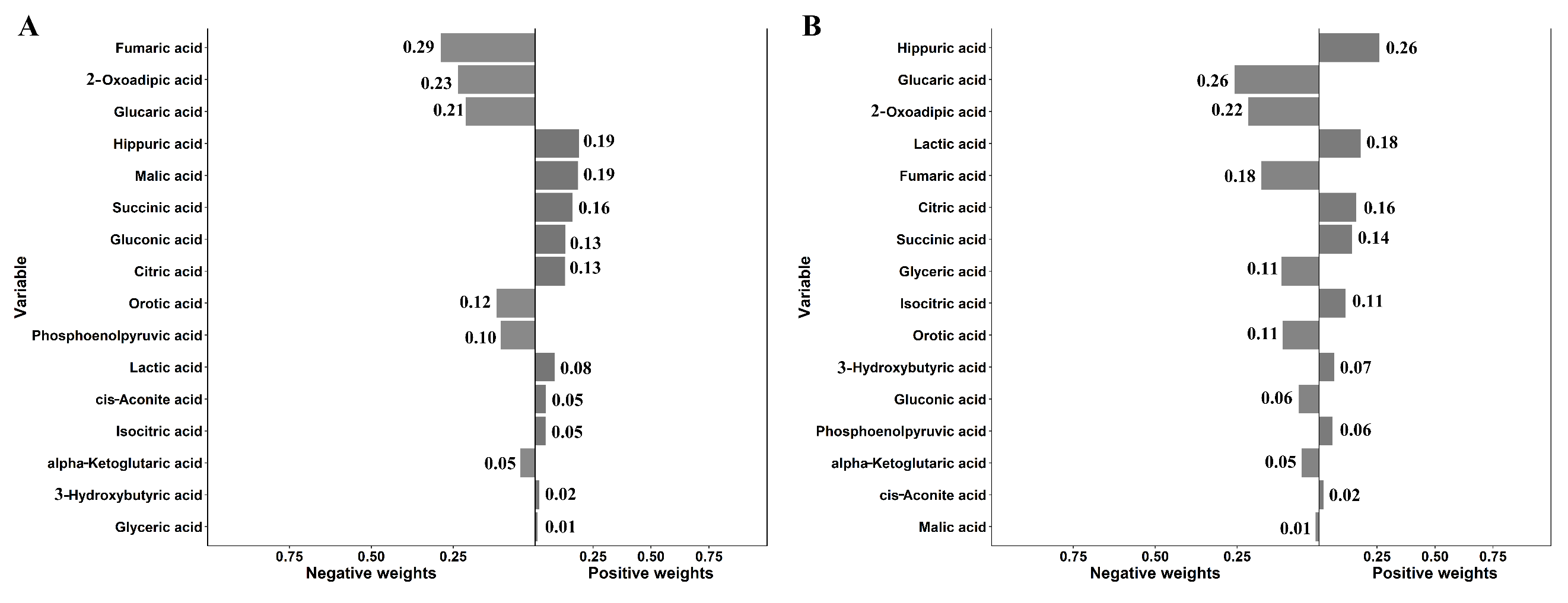
 **S5 Fig. Weights representing the proportion of the positive or negative partial effect for each metabolite in the quantile g-computation model with all metabolites in subgroups stratified by age.** (A) age < 65 years; (B) age ≥ 65 years.
