# Supplementary material for: Targeted metabolomics reveals the association between central carbon metabolism and pulmonary nodules
Source: PLoS One. 2023 Dec 7;18(12):e0295276. doi: 10.1371/journal.pone.0295276 (PMC10703222; doi:10.1371/journal.pone.0295276)
Supplement: S1 Table — (DOCX) [file pone.0295276.s007.docx]

| No. | Metabolites | CAS ID | Scanning ion | Retention time (min) |
| --- | --- | --- | --- | --- |
| 1 | alpha-Ketoglutaric acid | 328-50-7 | 198.0/73.0 | 7.67 |
| 2 | 2-Oxadipic acid | 3184-35-8 | 258.0/73.0 | 8.17 |
| 3 | 3-Hydroxybutyric acid | 300-85-6 | 191.0/147.0 | 4.92 |
| 4 | Gluconic acid | 526-95-4 | 333.0/73.0 | 9.81 |
| 5 | Phosphoenolpyruvic acid | 138-08-9 | 368.8/147.0 | 7.84 |
| 6 | Fumaric acid | 110-17-8 | 244.9/73 | 6.17 |
| 7 | Glyceric acid | 473-81-4 | 292.0/73.0 | 6.13 |
| 8 | Succinic acid | 110-15-6 | 247.1/72.9 | 5.97 |
| 9 | Hippuric acid | 495-69-2 | 206.0/105.0 | 8.94 |
| 10 | Citric acid | 77-92-9 | 273.1/73.0 | 9.01 |
| 11 | Malic acid | 97-67-6 | 244.9/146.9 | 7.13 |
| 12 | Glucaric acid | 87-73-0 | 333.1/143.0 | 9.86 |
| 13 | Orotic acid | 65-86-1 | 357.0/73.0 | 8.64 |
| 14 | L-Lactic acid | 79-33-4 | 191.2/147.1 | 4.23 |
| 15 | cis-Aconite acid | 585-84-2 | 375.0/147.0 | 8.63 |
| 16 | Isocitric acid | 320-77-4 | 273.0/73.0 | 9.01 |

**S1 Table. The targeted detection information of candidate metabolites by GC-MS/MS.**
