# Supplementary material for: Targeted metabolomics reveals the association between central carbon metabolism and pulmonary nodules
Source: PLoS One. 2023 Dec 7;18(12):e0295276. doi: 10.1371/journal.pone.0295276 (PMC10703222; doi:10.1371/journal.pone.0295276)
Supplement: S2 Table — (DOCX) [file pone.0295276.s008.docx]

**S2 Table. Baseline characteristics of high-risk populations before and after inverse probability weighting.**

|  | Before IPW |  |  | After IPW |  |  |
| --- | --- | --- | --- | --- | --- | --- |
|  | Non-screened  (n=1011) | Screened (n=1984) | SMD | Non-screened  (n=1041) | Screened  (n=1954) | SMD |
| **Demographics** |  |  |  |  |  |  |
| Age, year (mean (SD)) | 61.06 (7.14) | 62.96 (6.73) | 0.273 | 62.43 (7.16) | 62.35 (6.80) | 0.012 |
| Sex (n, %) |  |  | 0.131 |  |  | 0.038 |
| Male | 766 (5.8) | 1388 (70.0) |  | 758 (72.8) | 1388 (71.0) |  |
| Female | 245 (24.2) | 596 (30.0) |  | 283 (27.2) | 566 (29.0) |  |
| Education (n, %) |  |  | 0.120 |  |  | 0.067 |
| Primary and below | 267 (26.4) | 614 (30.9) |  | 306 (29.4) | 587 (30.0) |  |
| Junior high school | 407 (40.3) | 802 (40.4) |  | 400 (38.4) | 798 (40.8) |  |
| High school and above | 337 (33.3) | 568 (28.6) |  | 335 (32.2) | 569 (29.1) |  |
| Income, RMB (n, %) |  |  | 0.112 |  |  | 0.057 |
| <50000 | 304 (30.1) | 579 (29.2) |  | 317 (30.5) | 577 (29.5) |  |
| 50000~99999 | 498 (49.3) | 1071 (54.0) |  | 529 (50.8) | 1044 (53.4) |  |
| ≥100000 | 209 (20.7) | 334 (16.8) |  | 195 (18.7) | 333 (17.0) |  |
| BMI, kg/m^2^ (n, %) |  |  | 0.056 |  |  | 0.033 |
| <18.5 | 19 (1.9) | 38 (1.9) |  | 20 (1.9) | 38 (1.9) |  |
| 18.5~23.9 | 499 (49.4) | 981 (49.4) |  | 518 (49.8) | 969 (49.6) |  |
| 24.0~27.9 | 416 (41.1) | 785 (39.6) |  | 418 (40.2) | 770 (39.4) |  |
| ≥28.0 | 77 (7.6) | 180 (9.1) |  | 85 (8.2) | 177 (9.1) |  |
| **Lifestyle habits** |  |  |  |  |  |  |
| Smoking status (n, %) |  |  | 0.142 |  |  | 0.038 |
| Never smoker | 314 (31.1) | 703 (35.4) |  | 370 (35.5) | 660 (33.8) |  |
| Current smoker | 545 (53.9) | 929 (46.8) |  | 502 (48.2) | 961 (49.2) |  |
| Former smoker | 152 (15.0) | 352 (17.7) |  | 169 (16.2) | 333 (17.0) |  |
| Passive smoking (n, %) |  |  | 0.023 |  |  | 0.029 |
| No | 315 (31.2) | 639 (32.2) |  | 320 (30.7) | 628 (32.1) |  |
| Yes | 696 (68.8) | 1345 (67.8) |  | 721 (69.3) | 1326 (67.9) |  |
| Drinking (n, %) |  |  | 0.017 |  |  | 0.074 |
| No | 669 (66.2) | 1297 (65.4) |  | 709 (68.1) | 1262 (64.6) |  |
| Yes | 342 (33.8) | 687 (34.6) |  | 332 (31.9) | 692 (35.4) |  |
| Tea consumption (n, %) |  |  | 0.056 |  |  | 0.007 |
| No | 423 (41.8) | 885 (44.6) |  | 454 (43.6) | 860 (44.0) |  |
| Yes | 588 (58.2) | 1099 (55.4) |  | 587 (56.4) | 1094 (56.0) |  |
| Exposure to occupational hazards (n, %) |  |  | 0.167 |  |  | 0.002 |
| No | 805 (79.6) | 1439 (72.5) |  | 779 (74.8) | 1463 (74.9) |  |
| Yes | 206 (20.4) | 545 (27.5) |  | 262 (25.2) | 491 (25.1) |  |
| Exercise (n, %) |  |  | 0.195 |  |  | 0.003 |
| No | 516 (51.0) | 821 (41.4) |  | 467 (44.9) | 873 (44.7) |  |
| Yes | 495 (49.0) | 1163 (58.6) |  | 574 (55.1) | 1081 (55.3) |  |
| Cooking (n, %) |  |  | 0.129 |  |  | 0.004 |
| No | 392 (38.8) | 647 (32.6) |  | 358 (34.4) | 676 (34.6) |  |
| Yes | 619 (61.2) | 1337 (67.4) |  | 683 (65.6) | 1278 (65.4) |  |
| Thurification (n, %) |  |  | 0.036 |  |  | 0.007 |
| No | 928 (91.8) | 1801 (90.8) |  | 946 (90.9) | 1778 (91.0) |  |
| Yes | 83 (8.2) | 183 (9.2) |  | 95 (9.1) | 176 (9.0) |  |
| Occupational exposure to organic solvent (n, %) |  |  | 0.009 |  |  | 0.010 |
| No | 982 (97.1) | 1924 (97.0) |  | 1009 (96.9) | 1897 (97.1) |  |
| Yes | 29 (2.9) | 60 (3.0) |  | 32 (3.1) | 57 (2.9) |  |
| **Personal and family history of diseases** |  |  |  |  |  |  |
| Hypertension (n, %) |  |  | 0.107 |  |  | 0.030 |
| No | 529 (52.3) | 932 (47.0) |  | 518 (49.8) | 944 (48.3) |  |
| Yes | 482 (47.7) | 1052 (53.0) |  | 523 (50.2) | 1010 (51.7) |  |
| Diabetes (n, %) |  |  | 0.120 |  |  | 0.010 |
| No | 865 (85.6) | 1609 (81.1) |  | 856 (82.2) | 1613 (82.5) |  |
| Yes | 146 (14.4) | 375 (18.9) |  | 185 (17.8) | 341 (17.5) |  |
| Cancer (n, %) |  |  | 0.058 |  |  | 0.057 |
| No | 974 (96.3) | 1888 (95.2) |  | 1004 (96.4) | 1861 (95.2) |  |
| Yes | 37 (3.7) | 96 (4.8) |  | 37 (3.6) | 93 (4.8) |  |
| Family history of cancer (n, %) |  |  | 0.220 |  |  | 0.005 |
| No | 644 (63.7) | 1050 (52.9) |  | 592 (56.9) | 1106 (56.6) |  |
| Yes | 367 (36.3) | 934 (47.1) |  | 449 (43.1) | 848 (43.4) |  |
| Family history of lung cancer in any relatives (n, %) |  |  | 0.176 |  |  | 0.046 |
| No | 827 (81.8) | 1479 (74.5) |  | 814 (78.2) | 1489 (76.2) |  |
| Yes | 184 (18.2) | 505 (25.5) |  | 227 (21.8) | 465 (23.8) |  |
| Family history of lung cancer in first-degree relatives (n, %) |  |  | 0.163 |  |  | 0.034 |
| No | 833 (82.4) | 1504 (75.8) |  | 821 (78.9) | 1513 (77.4) |  |
| Yes | 178 (17.6) | 480 (24.2) |  | 220 (21.1) | 441 (22.6) |  |

Abbreviations: IPW, inverse probability weighting; SMD, standardized mean difference; BMI, body mass index.
