# Supplementary material for: Targeted metabolomics reveals the association between central carbon metabolism and pulmonary nodules
Source: PLoS One. 2023 Dec 7;18(12):e0295276. doi: 10.1371/journal.pone.0295276 (PMC10703222; doi:10.1371/journal.pone.0295276)
Supplement: S3 Table — (DOCX) [file pone.0295276.s009.docx]

**S3 Table. Adjusted odds ratios [95% confidence interval (CI)] for PNs in subgroups stratified by age based on the single-metabolite model.**

| Metabolites | Subgroup  (years) | Q1 | Q2 | Q3 | Q4 | p-trend |
| --- | --- | --- | --- | --- | --- | --- |
| alpha-Ketoglutaric acid | <65 | 1.00 | 1.59(1.10~2.29) | 1.38(0.95~1.98) | 1.52(1.06~2.18) | 0.063 |
|  | ≥65 | 1.00 | 1.01(0.68~1.50) | 1.35(0.90~2.03) | 1.28(0.85~1.93) | 0.107 |
| 2-Oxadipic acid | <65 | 1.00 | 0.41(0.29~0.58) | 0.42(0.29~0.60) | 0.49(0.35~0.70) | <0.001 |
|  | ≥65 | 1.00 | 0.45(0.30~0.67) | 0.32(0.22~0.48) | 0.46(0.31~0.69) | <0.001 |
| 3-Hydroxybutyric acid | <65 | 1.00 | 1.23(0.86~1.75) | 1.43(1.01~2.04) | 1.37(0.95~1.98) | 0.053 |
|  | ≥65 | 1.00 | 1.66(1.08~2.53) | 1.28(0.84~1.95) | 1.68(1.11~2.53) | 0.058 |
| Gluconic acid | <65 | 1.00 | 1.09(0.77~1.56) | 1.53(1.07~2.19) | 1.39(0.96~2.02) | 0.022 |
|  | ≥65 | 1.00 | 0.70(0.45~1.08) | 0.83(0.56~1.23) | 0.93(0.63~1.36) | 0.907 |
| Phosphoenolpyruvic acid | <65 | 1.00 | 0.72(0.50~1.05) | 0.99(0.69~1.42) | 1.39(0.97~1.99) | 0.018 |
|  | ≥65 | 1.00 | 0.77(0.51~1.17) | 1.39(0.94~2.07) | 1.54(1.05~2.27) | 0.004 |
| Fumaric acid | <65 | 1.00 | 0.81(0.57~1.15) | 0.96(0.69~1.35) | 0.85(0.60~1.21) | 0.552 |
|  | ≥65 | 1.00 | 0.82(0.54~1.23) | 0.91(0.60~1.38) | 0.95(0.63~1.42) | 0.990 |
| Glyceric acid | <65 | 1.00 | 1.05(0.73~1.52) | 1.31(0.91~1.89) | 1.45(1.01~2.11) | 0.025 |
|  | ≥65 | 1.00 | 0.91(0.61~1.36) | 1.23(0.82~1.84) | 0.94(0.63~1.42) | 0.867 |
| Succinic acid | <65 | 1.00 | 0.86(0.60~1.24) | 1.36(0.95~1.95) | 1.97(1.38~2.80) | <0.001 |
|  | ≥65 | 1.00 | 1.39(0.90~2.14) | 1.62(1.07~2.45) | 1.89(1.25~2.86) | 0.002 |
| Hippuric acid | <65 | 1.00 | 1.18(0.82~1.70) | 1.78(1.24~2.54) | 2.57(1.79~3.69) | <0.001 |
|  | ≥65 | 1.00 | 1.52(0.97~2.40) | 1.96(1.27~3.04) | 3.05(1.98~4.68) | <0.001 |
| Citric acid | <65 | 1.00 | 1.21(0.85~1.72) | 1.76(1.24~2.52) | 2.05(1.43~2.95) | <0.001 |
|  | ≥65 | 1.00 | 1.17(0.75~1.83) | 1.48(0.97~2.27) | 1.80(1.19~2.72) | 0.002 |
| Malic acid | <65 | 1.00 | 1.34(0.93~1.92) | 2.11(1.48~2.99) | 1.76(1.21~2.54) | <0.001 |
|  | ≥65 | 1.00 | 1.16(0.76~1.79) | 1.63(1.06~2.50) | 1.62(1.07~2.46) | 0.008 |
| Glucaric acid | <65 | 1.00 | 0.93(0.66~1.30) | 0.77(0.54~1.10) | 0.93(0.65~1.34) | 0.467 |
|  | ≥65 | 1.00 | 0.58(0.38~0.88) | 0.57(0.38~0.86) | 0.71(0.48~1.05) | 0.134 |
| Orotic acid | <65 | 1.00 | 0.43(0.30~0.62) | 0.45(0.31~0.64) | 0.55(0.38~0.78) | 0.002 |
|  | ≥65 | 1.00 | 0.44(0.29~0.66) | 0.35(0.23~0.52) | 0.60(0.41~0.89) | 0.004 |
| L-Lactic acid | <65 | 1.00 | 1.34(0.93~1.95) | 2.71(1.88~3.91) | 1.48(1.02~2.17) | 0.002 |
|  | ≥65 | 1.00 | 1.59(1.04~2.42) | 2.22(1.47~3.34) | 2.05(1.36~3.08) | <0.001 |
| cis-Aconite acid | <65 | 1.00 | 1.07(0.75~1.53) | 1.25(0.88~1.79) | 2.01(1.40~2.88) | <0.001 |
|  | ≥65 | 1.00 | 0.92(0.60~1.40) | 0.98(0.65~1.48) | 1.45(0.98~2.16) | 0.047 |
| Isocitric acid | <65 | 1.00 | 1.17(0.82~1.67) | 1.20(0.83~1.74) | 1.66(1.15~2.39) | 0.009 |
|  | ≥65 | 1.00 | 0.63(0.41~0.97) | 0.87(0.59~1.30) | 1.20(0.82~1.77) | 0.184 |

The Models were adjusted for sex, smoking status, drinking, exercise, occupational exposure to organic solvent and thurification.
