# Supplementary material for: Targeted metabolomics reveals the association between central carbon metabolism and pulmonary nodules
Source: PLoS One. 2023 Dec 7;18(12):e0295276. doi: 10.1371/journal.pone.0295276 (PMC10703222; doi:10.1371/journal.pone.0295276)
Supplement: S4 Table — (DOCX) [file pone.0295276.s010.docx]

**S4 Table. Adjusted odds ratios [95% confidence interval (CI)] for PNs in subgroups stratified by age based on the multi-metabolite model.**

| Metabolites | Q1 | Q2 | Q3 | Q4 | p-trend | p-FDR |
| --- | --- | --- | --- | --- | --- | --- |
| Age < 65 years |  |  |  |  |  |  |
| 2-Oxadipic acid | 1.00 | 0.27(0.16~0.44) | 0.23(0.14~0.38) | 0.27(0.17~0.44) | <0.001 | <0.001 |
| Gluconic acid | 1.00 | 2.24(1.37~3.67) | 3.00(1.81~4.97) | 2.40(1.43~4.02) | 0.116 | 0.116 |
| Fumaric acid | 1.00 | 0.72(0.48~1.09) | 0.73(0.48~1.11) | 0.32(0.18~0.56) | <0.001 | <0.001 |
| Succinic acid | 1.00 | 0.84(0.56~1.26) | 1.15(0.75~1.76) | 1.97(1.18~3.28) | 0.011 | 0.013 |
| Hippuric acid | 1.00 | 1.19(0.82~1.73) | 1.58(1.08~2.32) | 2.01(1.27~3.19) | <0.001 | <0.001 |
| Malic acid | 1.00 | 1.29(0.86~1.93) | 1.93(1.24~3.01) | 1.59(0.88~2.87) | 0.002 | 0.003 |
|  |  |  |  |  |  |  |
| Age ≥ 65 years |  |  |  |  |  |  |
| Hippuric acid | 1.00 | 1.52(0.97~2.40) | 1.96(1.27~3.04) | 3.05(1.98~4.68) | <0.001 | <0.001 |

The Models were adjusted for sex, smoking status, drinking, exercise, occupational exposure to organic solvent and thurification.
