# Supplementary material for: Targeted metabolomics reveals the association between central carbon metabolism and pulmonary nodules
Source: PLoS One. 2023 Dec 7;18(12):e0295276. doi: 10.1371/journal.pone.0295276 (PMC10703222; doi:10.1371/journal.pone.0295276)
Supplement: S5 Table — (DOCX) [file pone.0295276.s011.docx]

**S5 Table. Adjusted odds ratios [95% confidence interval (CI)] for PNs in subgroups stratified by sex based on the single-metabolite model.**

| Metabolites | Subgroup | Q1 | Q2 | Q3 | Q4 | p-trend |
| --- | --- | --- | --- | --- | --- | --- |
| alpha-Ketoglutaric acid | male | 1.00 | 1.09(0.79~1.51) | 1.30(0.94~1.78) | 1.16(0.84~1.60) | 0.244 |
|  | female | 1.00 | 1.84(1.14~2.97) | 1.30 (0.78~2.19) | 2.10(1.28~3.44) | 0.014 |
| 2-Oxadipic acid | male | 1.00 | 0.36(0.27~0.50) | 0.34(0.25~0.47) | 0.40(0.29~0.54) | <0.001 |
|  | female | 1.00 | 0.66(0.40~1.08) | 0.51(0.31~0.86) | 0.77(0.47~1.26) | 0.206 |
| 3-Hydroxybutyric acid | male | 1.00 | 1.55(1.12~2.14) | 1.34(0.97~1.85) | 1.37(0.99~1.88) | 0.157 |
|  | female | 1.00 | 0.99(0.61~1.62) | 1.41(0.87~2.29) | 1.96(1.17~3.29) | 0.005 |
| Gluconic acid | male | 1.00 | 0.95(0.70~1.30) | 1.16(0.85~1.61) | 1.11(0.80~1.52) | 0.323 |
|  | female | 1.00 | 0.81(0.46~1.43) | 1.30(0.81~2.07) | 1.36(0.85~2.17) | 0.103 |
| Phosphoenolpyruvic acid | male | 1.00 | 0.74(0.53~1.02) | 1.05(0.76~1.44) | 1.37(1.01~1.87) | 0.007 |
|  | female | 1.00 | 0.80(0.47~1.35) | 1.60(0.99~2.58) | 1.74(1.06~2.83) | 0.004 |
| Fumaric acid | male | 1.00 | 0.73(0.53~0.99) | 0.79(0.58~1.08) | 0.74(0.54~1.01) | 0.109 |
|  | female | 1.00 | 1.12(0.66~1.88) | 1.37(0.85~2.23) | 1.34(0.82~2.19) | 0.167 |
| Glyceric acid | male | 1.00 | 0.92(0.68~1.24) | 1.30(0.96~1.76) | 1.02(0.74~1.40) | 0.381 |
|  | female | 1.00 | 1.37(0.70~2.68) | 1.37(0.72~2.60) | 1.72(0.92~3.21) | 0.090 |
| Succinic acid | male | 1.00 | 1.04(0.76~1.44) | 1.58(1.16~2.16) | 1.89(1.38~2.59) | <0.001 |
|  | female | 1.00 | 1.08(0.64~1.82) | 1.20(0.71~2.04) | 1.86(1.13~3.06) | 0.100 |
| Hippuric acid | male | 1.00 | 1.15(0.83~1.61) | 1.95(1.41~2.68) | 2.69(1.95~3.72) | <0.001 |
|  | female | 1.00 | 1.84(1.08~3.15) | 1.59(0.93~2.70) | 2.80(1.68~4.67) | <0.001 |
| Citric acid | male | 1.00 | 1.41(1.02~1.96) | 1.86(1.34~2.57) | 2.04(1.47~2.83) | <0.001 |
|  | female | 1.00 | 0.85(0.51~1.43) | 1.25(0.76~2.06) | 1.78(1.09~2.90) | 0.009 |
| Malic acid | male | 1.00 | 1.25(0.90~1.73) | 1.94(1.41~2.68) | 1.58(1.14~2.18) | 0.001 |
|  | female | 1.00 | 1.26(0.76~2.09) | 1.56(0.95~2.58) | 1.91(1.16~3.15) | 0.007 |
| Glucaric acid | male | 1.00 | 0.87(0.63~1.21) | 0.66(0.48~0.90) | 0.76(0.56~1.05) | 0.035 |
|  | female | 1.00 | 0.59(0.37~0.95) | 0.93(0.56~1.55) | 1.07(0.66~1.72) | 0.591 |
| Orotic acid | male | 1.00 | 0.42(0.31~0.57) | 0.38(0.28~0.52) | 0.52(0.38~0.70) | <0.001 |
|  | female | 1.00 | 0.55(0.33~0.92) | 0.56(0.33~0.94) | 0.82(0.50~1.33) | 0.569 |
| L-Lactic acid | male | 1.00 | 1.46(1.06~2.02) | 2.45(1.78~3.36) | 1.54(1.10~2.15) | <0.001 |
|  | female | 1.00 | 1.46(0.85~2.51) | 2.25(1.34~3.78) | 1.91(1.17~3.12) | 0.004 |
| cis-Aconite acid | male | 1.00 | 0.99(0.72~1.36) | 1.12(0.82~1.54) | 1.61(1.18~2.20) | 0.002 |
|  | female | 1.00 | 0.98(0.59~1.63) | 1.13(0.68~1.89) | 2.02(1.22~3.32) | 0.005 |
| Isocitric acid | male | 1.00 | 0.93(0.67~1.29) | 1.09(0.79~1.49) | 1.43(1.04~1.96) | 0.013 |
|  | female | 1.00 | 0.99(0.62~1.58) | 0.97(0.58~1.62) | 1.59(0.98~2.59) | 0.092 |

The Models were adjusted for age, smoking status, drinking, exercise, occupational exposure to organic solvent and thurification.
