# Supplementary material for: Targeted metabolomics reveals the association between central carbon metabolism and pulmonary nodules
Source: PLoS One. 2023 Dec 7;18(12):e0295276. doi: 10.1371/journal.pone.0295276 (PMC10703222; doi:10.1371/journal.pone.0295276)
Supplement: S6 Table — (DOCX) [file pone.0295276.s012.docx]

**S6 Table. Adjusted odds ratios [95% confidence interval (CI)] for PNs in subgroups stratified by sex based on the multi-metabolite model.**

| Metabolites | Q1 | Q2 | Q3 | Q4 | p-trend | p-FDR |
| --- | --- | --- | --- | --- | --- | --- |
| **Male** |  |  |  |  |  |  |
| 2-Oxadipic acid | 1.00 | 0.26(0.17~0.39) | 0.22(0.14~0.34) | 0.23(0.16~0.35) | <0.001 | <0.001 |
| 3-Hydroxybutyric acid | 1.00 | 1.82(1.28~2.59) | 1.54(1.08~2.19) | 1.66(1.15~2.41) | 0.057 | 0.068 |
| Gluconic acid | 1.00 | 1.51(0.99~2.32) | 1.74(1.12~2.69) | 1.56(1.01~2.42) | 0.711 | 0.711 |
| Fumaric acid | 1.00 | 0.74(0.52~1.04) | 0.68(0.48~0.98) | 0.35(0.23~0.54) | <0.001 | <0.001 |
| Succinic acid | 1.00 | 1.26(0.89~1.79) | 1.81(1.27~2.58) | 2.52(1.65~3.84) | <0.001 | <0.001 |
| Hippuric acid | 1.00 | 1.12(0.79~1.58) | 1.65(1.18~2.32) | 1.90 (1.28~2.85) | <0.001 | <0.001 |
|  |  |  |  |  |  |  |
| **Female** |  |  |  |  |  |  |
| Hippuric acid | 1.00 | 1.83(1.07~3.13) | 1.58(0.93~2.70) | 2.75(1.65~4.60) | <0.001 | <0.001 |

The Models were adjusted for age, smoking status, drinking, exercise, occupational exposure to organic solvent and thurification.
