# Supplementary material for: Targeted metabolomics reveals the association between central carbon metabolism and pulmonary nodules
Source: PLoS One. 2023 Dec 7;18(12):e0295276. doi: 10.1371/journal.pone.0295276 (PMC10703222; doi:10.1371/journal.pone.0295276)
Supplement: Attachment — Submitted filename: Response to Reviewers.docx [file pone.0295276.s013.docx]

Dear Editor and Reviewers,

We are very pleased to hear from you about decision of our manuscript entitled “Targeted metabolomics reveals the association between central carbon metabolism and pulmonary nodules. (Manuscript Number: PONE-D-23-30022)”. We would like to express our sincere thanks to reviewers for thoughtful comments on the previous draft. We have carefully taken their comments into consideration in our revised manuscript, and reply to you immediately. The responses to reviewer’s comments are presented below.

**Reviewer #1:**

1-Abstract: The authors stated that “The aim of this study was to investigate the underlying mechanisms of PNs formation using metabolomics studies”. This statement is irrelevant to the current study as the present study has a cross-sectional design. Hence, the authors cannot provide a certain mechanistic insight for PN formation. Please rewrite it.

**Answer:** We have made the modifications in the Abstract.

2- The novelty of the current study cannot be presented well. It is highly recommended that the authors provide more explanation regarding the study's novelty in the “Introduction section”.

**Answer:** We have supplemented the novelty of this article in the “Introduction section”.

3- In statistical analysis, authors performed logistic regression models with the individual weights in models to estimate the weighted associations between central carbon metabolites and the risk of PNs. I was wondering why the authors selected triglycerides for model 3. Please elaborate on it.

**Answer:** It has been shown that lipid metabolism will be affected by the concentration of central carbon metabolites, and then regulate the triglyceride concentration in the body. The triglyceride is more effective after adjusting in the model three, which also verifies this point.

Thanks to your suggestions, should you need any further information, please feel free to contact us. We look forward to hearing from you.

Yours Sincerely,

Yueyang Wu

Wenbin Shen

Fen Huang

Qirong Qin
